# Nickel as a potential disruptor of thyroid function: benchmark modelling of human data

**DOI:** 10.3389/fendo.2023.1145153

**Published:** 2023-09-20

**Authors:** Djurdjica Maric, Katarina Baralic, Dragana Javorac, Stefan Mandic-Rajcevic, Milos Zarkovic, Biljana Antonijevic, Danijela Djukic-Cosic, Zorica Bulat, Aleksandra Buha Djordjevic

**Affiliations:** ^1^ Department of Toxicology “Akademik Danilo Soldatović”, University of Belgrade — Faculty of Pharmacy, Belgrade, Serbia; ^2^ School of Public Health and Health Management and Institute of Social Medicine, University of Belgrade – Faculty of Medicine, Belgrade, Serbia; ^3^ Department of Endocrinology, Diabetes and Metabolic Diseases, Belgrade, Serbia

**Keywords:** dose-response analysis, BMD modeling, toxic metals, endocrine disruptors, thyroid hormone level

## Abstract

**Introduction:**

Nickel (Ni) is one of the well-known toxic metals found in the environment. However, its influence on thyroid function is not explored enough. Hence, the aim of this study was to analyse the potential of Ni to disrupt thyroid function by exploring the relationship between blood Ni concentration and serum hormone levels (TSH, T4, T3, fT4 and fT3), as well as the parameters of thyroid homeostasis (SPINA-GT and SPINA-GD) by using correlation analysis and Benchmark (BMD) concept.

**Methods:**

Ni concentration was measured by ICP-MS method, while CLIA was used for serum hormone determination. SPINA Thyr software was used to calculate SPINA-GT and SPINA-GD parameters. BMD analysis was performed by PROAST software (70.1). The limitations of this study are the small sample size and the uneven distribution of healthy and unhealthy subjects, limited confounding factors, as well as the age of the subjects that could have influenced the obtained results.

**Results and discussion:**

The highest median value for blood Ni concentration was observed for the male population and amounted 8,278 µg/L. Accordingly, the statistically significant correlation was observed only in the male population, for Ni-fT4 and Ni-SPINA-GT pairs. The existence of a dose-response relationship was established between Ni and all the measured parameters of thyroid functions in entire population and in both sexes. However, the narrowest BMD intervals were obtained only in men, for Ni - SPINA-GT pair (1.36-60.9 µg/L) and Ni - fT3 pair (0.397-66.8 µg/L), indicating that even 78.68 and 83.25% of men in our study might be in 10% higher risk of Ni-induced SPINA-GT and fT3 alterations, respectively. Due to the relationship established between Ni and the SPINA-GT parameter, it can be concluded that Ni has an influence on the secretory function of the thyroid gland in men. Although the further research is required, these findings suggest possible role of Ni in thyroid function disturbances.

## 1 Introduction

Nickel (Ni) is one of the known toxic metals found in the environment. In nature, it is in the Earth’s crust in the form of various oxides or in combination with other metals. Due to its characteristics, primarily corrosion resistance, it is widely used in industry: production of alloys, electroplating, production of nickel-cadmium batteries, welding, it has also found application as a catalyst in the chemical and food industries (1). Nickel is present in food, air and water, while human exposure primarily comes from a large number of products that contain Ni (e.g. jewelry, coins, stainless steel, medical devices) (1, 2). For certain bacteria, plant and animal species, Ni is essential trace element, while this role of Ni in humans has not been elucidated yet (3). According to the Agency for Toxic Substances and Disease Registry (ATSDR) reference values for nickel in healthy adults is 0.2 µg/L in serum and 1–3 µg/L in urine. This metal has long been known to exhibit various toxic effects on human health. Some of the adverse effects associated with Ni exposure are on gastrointestinal, respiratory and cardiovascular system, kidneys, as well as contact dermatitis (4, 5). According to the International Agency for Research on Cancer (IARC), Ni and its compounds are classified in the first group of carcinogens, while the cancers they are connected with are lung, nasal cavity and paranasal sinus cancer. Also, there are studies linking exposure to Ni with the pancreatic cancer development. Twenty-eight patients with confirmed exocrine pancreatic cancer were included in the study, while control tissue came from deceased patients whose cause of death was not cancer. Criteria such as gender, age or cancer stage were not considered as criteria for excluding subjects from the study. The results of this study indicated higher Ni concentration in cancerous tissue was in comparison with the healthy one (6). Similar to many other toxic metals, Ni is considered to have the potential to cause endocrine disruption in humans. There are studies linking exposure to this metal and the increased prevalence of diabetes. The study included 2115 respondents, aged between 55 and 76 years, and the mean concentration of Ni in the urine was 3.63 mg/L. Elevated urinary Ni levels were associated with higher fasting glucose, glycated hemoglobin A1, insulin and homeostatic model assessment of insulin resistance. A relationship between increased urinary Ni concentration and increased prevalence of type 2 diabetes has been demonstrated (7). Some studies even show the potential influence of Ni on the function of many hormones such as growth hormone, prolactin, reproductive hormones, adrenal cortex hormones and adrenaline, but also thyroid hormones (8, 9).

The development of the nervous, circulatory, and reproductive system, as well as metabolic functions are all profoundly influenced by thyroid hormones (TH) (10). In the hypothalamic–pituitary–thyroid axis, hypothalamus secretes thyrotropin-releasing hormone (TRH) and controls TSH production which induces the production of T4 and T3 in thyroid cells. These hormones, when present in the blood, further modulate the production and release of TSH (8). Nowadays, thyroid disorders are a widespread issue, that can remarcably reduce life quality (11). The incidence of hypothyroidism, most frequently caused by Hashimoto’s thyroiditis, is between 1 and 2% in locations with no marked iodine deficit, while women are 10 times more prone to developing this condition than men. The incidence of hyperthyroidism in women is between 0.5 and 2%, and it additionally affects them more frequently than men, while it is estimated that between 10 and 14 percent of people in the general population have subclinical hypothyroidism (12). Although the exact reasons of thyroid gland malfunction are unknown, it is recognized that a number of toxic metals, including Ni, that are ubiquitous in the environment, can promote the emergence of these conditions. However, to the best of our knowladge, the influence of Ni on development of thyroid diseases is not explored enough, while the precise molecular mechanisms by which Ni affects thyroid function are unknown.

In a recent animal study, after intraperitoneal NiSO_4_ administration, there was a significant decrease in the concentration of serum fT4 and TSH in male rats for all the tested dose levels (2.5, 5 and 10 mg/kg day, during 40 days, intraperitoneally), and direct damage of the thyroid cells caused by Ni was proposed as one of the most important mechanisms, more precisely, apoptosis closely related to alternations of Caspase-3, Bcl2, and Fas mRNA and protein expression (13). The relationship between Ni and thyroid hormones imbalance was also investigated in humans. However, in a study involving Chinese pregnant women, the linkage between blood Ni concentration and TH level was not confirmed (14). The other authors have, on the other hand, suggested the potential interaction of Ni with other toxic metals, mainly mercury (Hg), in the occurrence of toxic effects at the level of the thyroid gland (15), while double prevalence of chronic immune thyroiditis was noted in patients with Ni allergy (16).

The benchmark dose (BMD) method can be employed to determine the relationship between the dosage of the investigated chemical and the effect. For the purpose of risk assessment, data from animal studies are mostly available, and in a smaller number of cases, data from epidemiological studies are used. Of course, the use of data from epidemiological studies has its advantages because it is not necessary to extrapolate data from animals to humans, but it also carries uncertainties due to a large number of factors that cannot be controlled. Formerly, the principle of the No Observed Adverse Effect Level (NOAEL) was used to accomplish this goal. In comparison to the NOAEL technique, BMD modeling allows greater data utilization and eliminates the effect of experimental design on the research (17). The European Food Safety Agency (EFSA) recognizes this idea as crucial in epidemiological investigations. However, the number of epidemiological studies in which BMD modeling is used is still restricted. This method has been employed successfully in the interpretation of human study data in some of our previously published articles (18–22). For example, we have demonstrated the existence of a dose-response relationship between mercury (Hg) in blood and all the measured thyroid hormones, with the most accurate modeling prediction for the effects on TSH in women, suggesting that even values greater than 0.626 µg/L could cause the increase of TSH in women (23).

SPINA technique (structure parameter inference approach) uses serum/plasma hormone levels to estimate the parameters of endocrine feedback control mechanisms. This is a new technique that has proven successful in evaluating thyroid function (24). SPINA Thyr is a diagnostic application, and the SPINA Thyr algorithm has been evaluated in several clinical trials on a numerous patients. The usefulness of SPINA parameters in the diagnosis of thyroid gland disease has been proven (25, 26). To calculate these parameters, laboratory-determined TSH, fT4 and fT3 values are used, which is the main advantage of applying this model, because observing only hormone levels in the blood is often not enough to confirm thyroid function disorders. The two main parameters used to assess thyroid function are SPINA-GT and SPINA-GD. SPINA-GT is a parameter which describes the production of T4. It is the amount of T4 that is released per unit of time at maximum stimulation of the gland with TSH. In addition to TSH and fT4 levels, kinetic parameters and plasma protein binding parameters are included in the calculation of this parameter. SPINA-GD is a parameter which describes the activity of peripheral deiodinases, and its calculation includes fT3 and fT4 as well as certain constants (25, 27). In a previous study, SPINA-GT was compared in healthy subjects and patients with various thyroid disorders. The result showed a significant increase in the SPINA-GT parameter in patients with Graves’ Disease, toxic adenoma and goiter, as well as a significant decrease in autoimmune thyroiditis. Additionally, the greater specificity of this parameter for hyperthyroidism in toxic adenoma compared to hormones (TSH, fT3 and fT4) was demonstrated (25).

Having all of this in mind, the aim of this study was to analyze the potential of Ni to disrupt thyroid function by exploring the relationship between Ni concentration in the subjects’ blood and serum hormone levels (TSH, T4, T3, fT4 and fT3), as well as the parameters of thyroid homeostasis (SPINA-GT and SPINA-GD) by using correlation analysis and BMD concept.

## 2 Matherial and methods

### 2.1 Study population

Population characteristics and study design are presented in the **Table 1**.

**Table 1:** Population characteristics and study design.

*Population*	Subjects from the general population at the Clinical Center of Serbia and the Clinical Hospital Center “Bežanijska kosa” in Belgrade, Serbia: • healthy volunteers (136) • prostate/testicular cancer patients (104) • breast cancer and benign breast dysplasia patients (96) • pancreatic cancer patients (22) • thyroid and metabolic disorders patients (77)*
*Number of participants*	Total number: 435 subjects (218 women and 217 men)
*Age of the participants*	between 18 and 94 years * average age for women was 50 years, and for men 51 years * average age for healthy population was 43 years, and for unhealthy population 55 years
*Ethics*	The study was conducted in line with the Helsinki Declaration’s ethical obligations.
*Ethical approvals*	o University Hospital “Bežanijska kosa” Medical Center’s Scientific and Ethical Committee (9740/3) o Clinical Center of Serbia’s Ethical Committee (526/9, 31/8, and 579/19) o Medical Faculty of the University of Belgrade (1322/XII-5) o University of Belgrade’s Ethical Commission for Biomedical Research (650/2 and 288/2)
*Sample*	Blood (K_2_EDTA) – metal analysis Serum – hormone analysis

*To assess correlations and dose-response relationships between Ni levels and parameters of thyroid function in the whole examined population, all groups were taken into account collectively and later referred to as the “general population” throughout all stages of our investigation.

### 2.2 Determination of Ni, thyroid hormone concentrations and parameters of thyroid homeostasis

Following blood clotting, serum was separated by centrifugation at 3000 x g for 30 minutes for hormone analysis. In order to prepare EDTA-blood for Ni detection, 1 ml of blood was digested in a microwave oven with 7 ml of 65% HNO_3_ and 1ml of 30% H_2_O_2_ (Milestone START D, SK-10T, Milestone Srl, Sorisole, Italy). Blank, which underwent the same procedure as all of the samples, consisted of the used reagents (7 ml of 65% HNO_3_ and 1 ml of 30% H_2_O_2_). The following program was applied: heating (15 min at 180°C), digestion (15 min at 180°C), and cooling. Ni was determined using the ICP-MS technique (ICP-MS 7700, Agilent Technologies, Santa Clara, CA, USA). For calibration, an external standard approach [multielement standard solution 1 g/L in diluted nitric acid (Merck, Darmstadt, Germany)] was used. The accuracy of the method was tested by the standard reference material (Seronorm TM, Sero, Billingstad, Norway). When determining Ni concentration, the trueness varied from 91.9–106.2%, while the limit of detection (LoD) 0.0255 μg/L, and the number of replication is 3.

The chemiluminescent immunoassay (CLIA) method was employed on the Liason series of analyzers to assess the levels of hormones (TSH, fT4, fT3, T4, T3) (DiaSorin Inc, USA). When determining hormone levels, the LOD and LOQ were as follows: TSH – LOD: 0.005 µIU/ml, LOQ: 0.005 µIU/ml; fT4 – LOD: 0.5 pmol/L, LOQ: 3 pmol/L; fT3 – LOD: 0.6 pmol/L, LOQ: 1.5 pmol/L; T4 – LOD: 5.4 nmol/L, LOQ: 15 nmol/L; T3 – LOD: 0.3 nmol/L, LOQ: 0.3 nmol/L. Thyroid homeostasis parameters, SPINA-GT and SPINA-GD, were derived based on calculation method provided by Dietrich et al. (2016) (25).

### 2.3 Statistical analysis

SPINA Thyr software was used to calculate SPINA-GT and SPINA-GD parameters. In order to calculate these parameters, the levels of TSH, fT3 and fT4, which were determined in the serum of our population, were needed. Below are the equations that describe how these parameters are calculated. **Table 2** shows the values of the constants used in the calculation (27, 28).

**Table 2:** Parameters used in SPINA calculation.

Symbol	Explanation	Value
αT	Dilutin factor tor thyroxine	0.1 L^-1^
βT	Clearance exponent for T4	1.1e-6 s^-1^
DT	EC_50_ for TSH	2.75 mIU/L
K41	Dissociation constant of T4 at thyroxine-binding globulin	2e10 L/mol
K42	Dissociation constant of T4 at transthyretin	2e8 L/mol
α31	Dilution factor for triiodothyronine	0.026 L^-1^
β31	Clearance exponent for T3	8e-6 s^-1^
KM1	Dissociation constant of type 1 deiodinase	500 nmol/L
K30	Dissociation constant of T3 at thyroxine-binding globulin	2e9 L/mol
[TBG]	Standard concentration of thyroxine-binding globulin	300 nmol/L
[TBPA]	Standard transthyretin concentration	4.5 µmol/L
β	Correction coefficient of logarithmic model	0.1345

The SPINA-GT parameter is calculated according to the equation:


$$GT=\frac{\beta T\;\left(DT+\;\left[TSH\right]\right)\left(1+K41\;\left[TBG\right]+K42\left[TBPA\right]\right)\left[fT4\right]\;}{\alpha T\left[TSH\right]}$$

The SPINA-GD parameter is calculated according to the equation:


$$GD=\frac{\beta 31\;\left(KM1+\;\left[fT4\right]\right)\left(1+K30\;\left[TBG\right]+\left[TBG\right]\right)\left[fT3\right]\;}{\alpha 31\left[fT4\right]}$$

Data were analyzed using Graph Pad Prism 8 software (GraphPad Software Inc., San Diego, USA). After testing the normality of the distribution, homogeneity and variance, Spearman’s correlation analysis was performed to examine the relationship between the measured blood Ni concentration and thyroid hormone levels (TSH, fT4, fT3, T4, T3)/parameters of thyroid function (SPINA-GT, SPINA-GD).

For dose-response analysis, PROAST software (70.1) was utilized (the Dutch National Institute for Public Health and the Environment, RIVM) to establish the relationships between blood Ni level and serum thyroid hormone concentrations.

Following settings were applied:

o Data type – quantal [0: within the reference range (TSH: 0.270-4.20 µIU/ml, fT4: 12-22 pmol/L, fT3: 3.1-6.8 pmol/L, T4: 66-181 nmol/L, T3: 1.3-3.1 nmol/L; SPINA-GD: 20-60 nmol/s, SPINA-GT: 1.4-8.7 pmol/s); 1: outside of the reference range]
o Covariant: gender
o The BMD interval (BMDI) determination: model averaging method (200 bootstrap iterations), Akaike information criterion
o BMR: 10% increased risk of thyroid hormone disturbances

## 3 Results

**Table 3** shows the subjects classified according to whether their values of the investigated hormones and parameters were within the reference range or not.

**Table 3:** The examined population in relation to gender and the values of the examined parameters (in or outside the reference range).

Subjects Parameter	In reference range		Out of reference range	
	Male	Female	Male	Female
TSH (µIU/ml)	168	177	25	26
fT4 (pmol/L)	165	176	20	32
fT3 (pmol/L)	150	184	38	20
T4 (nmol/L)	189	197	7	9
T3 (nmol/L)	182	174	12	27
SPINA-GT (pmol/s)	120	180	63	22
SPINA-GD (nmol/s)	120	179	64	25

**Table 4** represent the descriptive statistics, including median, 25 and 75 percentiles of the measured blood Ni concentration, serum levels of thyroid hormones and calculated parameters of thyroid function in the general population from Belgrade, Republic of Serbia. The values are presented separately for male and female, but also for the entire population.

**Table 4:** Descriptive statistic including median values, 25 and 75 percentiles of measured blood Ni concentration, serum levels of thyroid hormone and calculated parameters of thyroid function.

	Parameter	Ni (µg/L)	TSH (µIU/ml)	fT4 (pmol/L)	fT3 (pmol/L)	T4 (nmol/L)	T3 (nmol/L)	SPINA-GD (nmol/s)	SPINA-GT (pmol/s)
Men	25% Percentile	2,902	1,020	15,37	4,295	101,4	1,630	2,559	22,93
	Median	8,278	1,870	16,84	4,760	116,5	1,950	3,286	26,30
	75% Percentile	14,20	2,700	19,09	5,290	132,8	2,180	4,932	29,44
Women	25% Percentile	1,771	1,105	5,718	4,173	94,66	1,658	1,238	24,47
	Median	7,609	1,790	15,63	4,910	109,7	1,955	2,783	29,18
	75% Percentile	15,70	2,530	18,46	5,623	125,0	2,203	4,225	59,08
Entire population	25% Percentile	2,428	1,083	14,24	4,245	97,97	1,640	2,158	23,70
	Median	8,054	1,810	16,54	4,830	113,2	1,950	3,064	26,98
	75% Percentile	15,46	2,628	18,77	5,440	129,9	2,183	4,551	32,09

After Spearman’s correlation analysis (Ni vs. thyroid hormone levels/thyroid function parameters determined in blood samples from male, female, and the entire examined population of Belgrade, Serbia), (**Tables 5**, **6**), out of the all the compared parameters, correlation was observed only for Ni and fT4 and Ni and SPINA-GT pairs in males. In female population and entire population, no correlation was observed between Ni and neither of the measured/calculated parameters.

**Table 5:** Results of Spearman’s correlation analysis between Ni and thyroid hormone levels in blood samples from male, female, and the entire examined population of Belgrade, Serbia.

		Ni vs. TSH	Ni vs. fT4	Ni vs. fT3	Ni vs. T4	Ni vs. T3
Men	r	0,03853	-0,1556	-0,07936	0,02239	0,09980
	95% confidence interval	-0,1114 to 0,1867	-0,3007 to -0,003492	-0,2280 to 0,07288	-0,1265 to 0,1703	-0,04982 to 0,2450
	P value	0,6046	**0,0391**	0,2923	0,7623	0,1777
Women	r	0,02065	0,01262	-0,04637	0,06305	0,03597
	95% confidence interval	-0,1316 to 0,1719	-0,1377 to 0,1624	-0,1964 to 0,1057	-0,08874 to 0,2120	-0,1178 to 0,1880
	P value	0,7850	0,8661	0,5388	0,4018	0,6375
Entire population	r	0,03338	-0,07270	-0,07534	0,04009	0,06921
	95% confidence interval	-0,07327 to 0,1393	-0,1782 to 0,03441	-0,1809 to 0,03191	-0,06600 to 0,1453	-0,03776 to 0,1746
	P value	0,5278	0,1705	0,1560	0,4457	0,1914

A significant result is marked in red.

**Table 6:** Results of Spearman’s correlation analysis between Ni and thyroid function parameters (SPINA-GT and SPINA-GD) from male, female, and the entire examined population of Belgrade, Serbia.

		Ni vs. SPINA-GT	Ni vs. SPINA-GD
Men	r	-0,1889	0,1328
	95% confidence interval	-0,3324 to -0,03684	-0,02074 to 0,2802
	P value	**0,0126**	0,0807
Women	r	0,02035	-0,07374
	95% confidence interval	-0,1323 to 0,1720	-0,2226 to 0,07850
	P value	0.7886	0.3279
Entire population	r	-0,09584	0,03374
	95% confidence interval	-0,2017 to 0,01219	-0,07413 to 0,1408
	P value	0,0733	0,5281

A significant result is marked in red.

The PROAST program was used to assess the dose-response relationship for male, female, and the entire examined population. The final results of BMD analysis are shown in **Tables 7**, **8**, presented as BMD intervals (BMDL and BMDU values). The dose-response relationship was established for all of the Ni-thyroid hormone levels/thyroid function parameters pairs. BMDL and BMDU represent the lower and the upper 95% confidence limit of the Benchmark dose, respectively. In addition to assessing which of the obtained BMDL values is the lowest, the width of the BMD interval (BMDI), the ratio between BMDL and BMDU, should be considered, having in mind that it indicates the reliability of the obtained results. In the present study, the lowest BMDL was obtained for the Ni - SPINA-GD pair in the overall population, but the BMDI was quite wide, indicating a lower degree of confidence in the prediction. In general, for the interval to be considered narrow, the ratio between BMDL and BMDU values should be less than 10 (29). In our study, the lowest ratio was obtained for Ni - SPINA-GT pair (1.36-60.9 µg/L) and for Ni - fT3 pair (0.397-66.8 µg/L). Additionally, for these two highlighted narrowest Ni - hormone/parameter pairs, the percentage of the population which was above the obtained BMDL values was calculated. 78.68% of men had Ni concentration higher than 1.36 µg/L, while 83.25% of the men had Ni concentration higher than 0.397 µg/L.

**Table 7:** Findings of the Benchmark dose-response analysis for Ni and thyroid hormone levels determined in blood samples from male, female, and the entire examined population of Belgrade, Serbia (PROAST web 70.1, https://proastweb.rivm.nl).

Values Parameter	Men		Women		Entire population	
	BMDL	BMDU	BMDL	BMDU	BMDL	BMDU
**TSH (µIU/ml)**	6.9	539000	1.83	7190000	5.58	6790000
**fT4 (pmol/L)**	6.31	830000	18	5110000	5.01	1530000
**fT3 (pmol/L)**	**0.397**	**66.8**	6.6	1690000	0.459	2480000
**T4 (nmol/L)**	99.1	675000	129	2460000	217	2130000
**T3 (nmol/L)**	33.4	586000	3.83	4000000	21.6	5130000

A significant result is marked in red.

**Table 8:** Findings of the Benchmark dose-response analysis for Ni and thyroid function parameters (SPINA-GT and SPINA-GD) from male, female, and the entire examined population of Belgrade, Serbia (PROAST web 70.1, https://proastweb.rivm.nl).

Values Parameter	Men		Women		Entire population	
	BMDL	BMDU	BMDL	BMDU	BMDL	BMDU
**SPINA-GT (pmol/s)**	**1.36**	**60.9**	4.25	2360000	0.00161	2970000
**SPINA-GD (nmol/s)**	0.00321	186	1.37	1470000	0.00137	972000

A significant result is marked in red.

Given that our population included healthy subjects and subjects suffering from various diseases, in order to examine the influence of Ni concentration on thyroid gland function, the dose-response relationship between Ni concentration and parameters of thyroid gland function (SPINA-GT and SPINA-GD) in healthy and sick subjects in particular. **Table 9** shows the obtained BMDL and BMDU values. A dose-response relationship was established in both healthy and unhealthy subjects, but the obtained intervals were wide.

**Table 9:** Findings of the Benchmark dose-response analysis for Ni and thyroid function parameters (SPINA-GT and SPINA-GD) from healthy/unhealthy subjects in entire examined population of Belgrade, Serbia (PROAST web 70.1, https://proastweb.rivm.nl).

Entire population Parameters	Healthy		Unhealthy	
	BMDL	BMDU	BMDL	BMDU
**SPINA-GT (pmol/s)**	0.0426	245	19.6	5.39e+17
**SPINA-GD (nmol/s)**	0.00683	580000	0.0408	3020000

**Figure 1:**
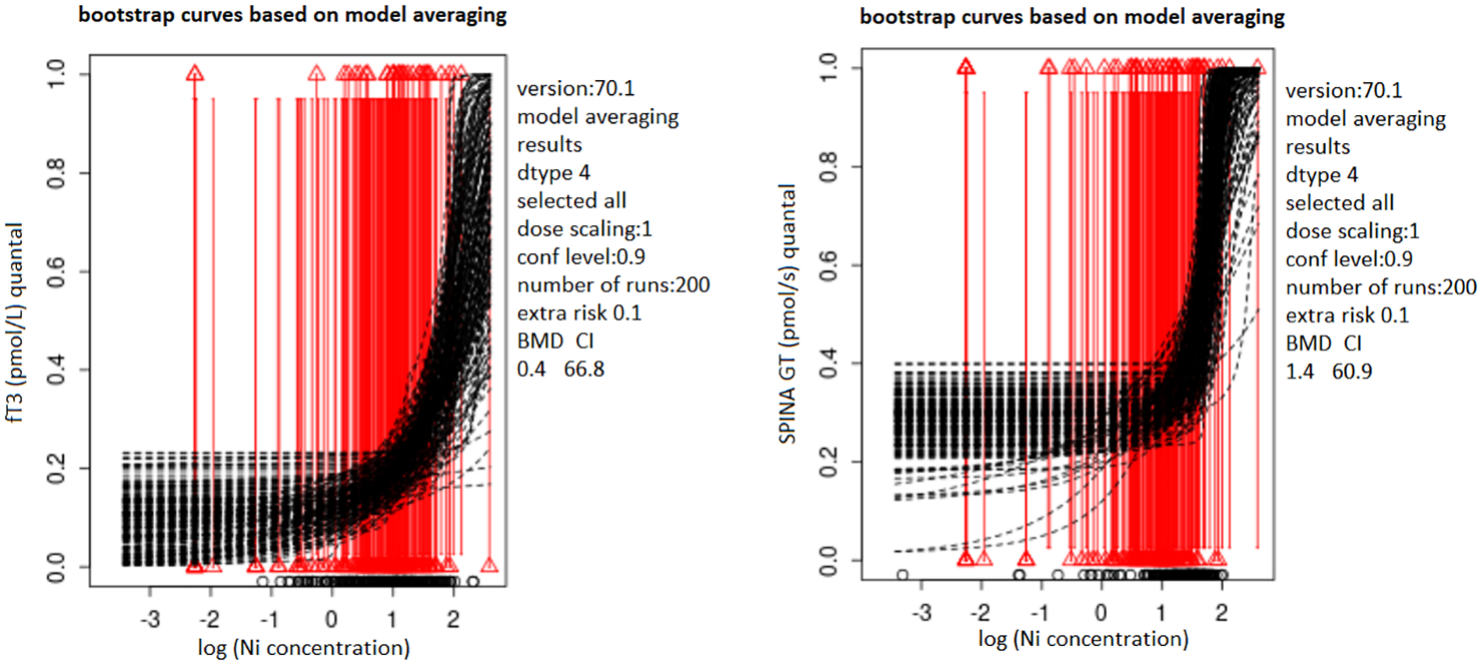
Bootstrap curves based on model averaging describing the dose-response relationship of Ni with fT3 **(A)**/SPINA-GT **(B)** in blood samples from the male population of Belgrade, Serbia; PROASTweb 70.1 software, https://proastweb.rivm.nl; quantal BMR of 10%. Black dashed lines represent bootstrap curves obtained based on model averaging process (200 bootstrap iteration). Red vertical lines represent quantal response, while triangles at the end of the lines indicate whether the response is within/out of the reference range (0 or 1). The x-axis represents log10 of Ni blood levels (µg/L) while the y-axis represents fT3 (pmol/L) and SPINA-GT (pmol/s) parameters presented as quantal values (within/out of the range).

Dose-response curves for Ni-fT3 and Ni-SPINA GT pairs, for which narrow BMDI intervals were obtained, based on model averaging in PROAST software are shown in **Figure 1**. In the BMD modeling procedure, model averaging was performed in 200 iterations using the bootstrap method and the Akaike information criteria (AIC). In this method, the results of several models are integrated and evaluated based on the model fit (30).

## 4 Discussion

Most toxicological studies are conducted on animals in which high doses of test substances are administered. Such an experiment cannot give reliable results when it comes to real exposure to environmental chemicals. This can be especially noted in the case of EDCs because of their characteristic to provoke intense effects even at very low doses and exert nonmonotonic behavior (31–33). In addition, the limitations of animal studies imply the application of one substance, by one route of exposure, often in a short time interval, and all of this makes it difficult to assess the risk of EDC exposure (34, 35).

The current investigation was conducted to examine the effect of Ni present in the blood of the general population on thyroid function. Hormones (TSH, fT4, fT3, T4 and T3) and thyroid function parameters (SPINA-GT and SPINA-GD) were examined. Serum levels of all of these hormones are considered the important measures of thyroid function, together with the calculated parameters, SPINA-GT, which represents the secretion capability of the thyroid gland, and SPINA-GD, which represents cumulative activity of peripheral deiodinases (25).

The connection between Ni and production of thyroid hormones at the level of the hypo-thalamus–pituitary–target gland axis was suggested. As the main mechanisms, perturbation in the levels of different apoptosis-related proteins, such as caspase-3, B-cell lymphoma 2, and Fas, in thyroid tissue have been suggested (13). Another suggested mechanism was oxidative stress, including the ability of Ni to decrease the reduced glutathione, non-protein thiol contents, as well as the activity of glutathione peroxidase and superoxide dismutase, but also increase the malondialdehyde content in thyroid gland tissue (36).

In the present study, the median value of the Ni blood concentration was 8.278, 7.609, and 8.054 µg/L for the male, female, and entire population, respectively. In a study conducted in Tunisia, the median Ni level in the blood of subjects from the general population was 22.99 µg/L (37), which is higher compared to the median Ni level in the blood of subjects in our country. However, compared to a study conducted in Germany in 2006 which assessed trace element levels in the general population of northern Germany, the estimated 5th–95th percentile was 0.03–0.22 µg/L (38), which is considerably lower than in the investigated Serbian population. It should also be noticed that median Ni level in men was the highest among the tested populations (8,278 µg/L), while it was slightly lower in women (7,609 µg/L). Regarding the thyroid hormone levels, TSH, fT4 and T4 were higher in men, while fT3 and T3 were higher in women. These differences might be attributed to hormonal differences in sexes in general, metabolic rate, body composition, lifestyle factors, etc.

In our study, no correlation was observed for Ni concentration and hormone levels and function parameters in the total population and the female population, while in the male population a negative correlation was observed for Ni and the measured level of serum fT4, as well as for Ni and SPINA-GT, indicating that higher levels of Ni could be connected with lower thyroid secretory capacity and, thus, lower fT4 levels in blood. Accordingly, the highest median value for blood Ni concentration was observed for male population (8,278 µg/L).

There is a small number of studies, especially epidemiologic, that have investigated the effects of Ni on thyroid hormones and thyroid function. Given that the frequency of these disorders is high and represents a global problem, examining additional risk factors may be of great importance for understanding their nature. A study including 110 subjects conducted in Iran aimed to link exposure to various metals, including Ni, to thyroid disorders. However, the results of this study have indicated that Ni levels were similar in healthy and unhealthy subjects (39). In the previously mentioned study including Chinese pregnant women, there was also no significant relationship between Ni levels and TH concentrations (14). Other authors have also tried to establish a link between exposure to Ni, but also other toxic metals, and thyroid disorders. For example, the authors emphasize the potential interaction of Ni with Hg in the occurrence of toxic effects at the level of the thyroid gland and the need for further research into the impact of this metal on thyroid function (15). Our previous study also established a link between Hg exposure and thyroid dysfunction (23). Elucidation of the precise mechanisms by which Ni and Hg may contribute to this disorder could indicate interactions if they exist. The relationship between Ni and thyroid gland function was investigated in a retrospective study in which the relationship between systemic allergic syndrome to Ni and chronic autoimmune thyroiditis, the most common cause of hypothyroidism in the areas where there is no iodine deficiency, was observed. Volunteers suspected of having immune-mediated inflammatory diseases were included in the research, and the analyses determined that the subjects who suffered from systemic allergic syndrome were exposed to higher Ni concentrations. As a conclusion, this study indicated a double prevalence of chronic immune thyroiditis in patients with Ni allergy (16).

In present study, in addition to correlation analysis, we performed the Benchmark approach, which is not widely used in epidemiological studies, but which proved to be valuable in this type of analysis. Our approach views reference values of thyroid hormones/thyroid function parameters were as a threshold to distinguish normal values from the ones which fell outside the range, while Ni exposure is viewed as one of the risk factors connected to these disorders. BMD analysis may be applied on human data in order to assess the link between the measured toxic agent in different biological samples and effects. For example, a study that applied the BMD principle in the context of human exposure aimed to uncover the link between cadmium (Cd) concentration and renal failure (40). Furthermore, Lin et al. (2018) derived the significant dosage for lead (Pb)-induced kidney impairment from a study that included individuals occupationally exposed to Pb using this concept (41). Finally, in a study based on NHANES data from 1999 to 2006, scientists have established a link between urinary Cd and type 2 diabetes mellitus (42).

Although the number of studies including this methodology is small, our previous research has supported the use of the BMD approach in the analysis of human data. In a study that examined the dose-response relationship between Cd, arsenic (As), Hg, Ni, and chromium (Cr) in the blood and testosterone, luteinizing, and follicle-stimulating hormones in the serum of men, a dose-response relationship was established for all metal-hormone pairs (19), and also for metal and testosterone in women, and estradiol and progesterone in men (22). In a study where the relationship between the exposure to Pb and endocrine function of the organism was monitored, the narrowest BMDI was obtained for the Pb-insulin pair (20). These studies point to two important conclusions. Firstly, the levels of toxic metal(oid)s in the general population might increase the risk of various endocrine disorders and secondly, use of BMD approach in studying human data has been shown as a valuable addition to standard statistical tests used so far to analyze the data sets obtained from epidemiological studies.

In the current study, a dose-response relationship was established for Ni and all the investigated thyroid function parameters. However, almost all of the obtained Ni-hormone/parameter pairs were wide. Relatively narrow intervals were obtained for Ni - fT3 (0.397-66.8 µg/L) and Ni - SPINA-GT (1.36-60.9 µg/L) in the male population, indicating higher accuracy in the estimation. Nevertheless, these intervals point to various sources of uncertainty which is a general issue when working with human data sets. The calculated median value for blood Ni concentration in men (8,278 µg/L) is within both intervals and is greater than the BMDL value (0.397 or 1.36 µg/L). Having in mind the measured blood Ni concentrations in the male population, the results of our study have indicated that 83.25% of men might have 10% higher risk of Ni-induced fT3 alterations, while this percentage was 78.68 for Ni-induced SPINA-GT alterations. The results obtained when the health status was considered as a variable indicate that a healthy population is more sensitive to Ni exposure which can be explained by the fact that in unhealthy participants various compensatory mechanisms have already been activated.

The fact that a narrow BMDI was obtained for the pair Ni - SPINA-GT has greater significance in the assessment of Ni influence on thyroid function. The importance of the SPINA-GT parameter for the evaluation of thyroid function was highlighted in a study involving 20 healthy subjects, where this parameter showed greater reliability compared to hormones which reflect acute regulation of function (TSH, fT3 and fT4). The authors concluded that SPINA-GT can be considered a constant parameter of thyroid homeostasis (27). The importance that SPINA-GT has in the evaluation of thyroid function may indicate even greater reliability of our result that Ni can disrupt the secretory function of the thyroid gland in men.

As previously mentioned, when assessing calculated data, several of the resulting BMD intervals were rather wide, showing a significant uncertainty level. Moreover, given that a variety of factors might affect hormone serum levels and overall thyroid function, it is difficult to judge whether the slight perturbances in hormone levels outside the reference range are always biologically significant. However, the relationship obtained between Ni and the SPINA-GT parameter is proof of the influence of Ni on the secretory thyroid gland function. However, it is important to consider that not merely environmental factors, but also inherited ones, might also influence the changes in thyroid hormone levels and, subsequently, changes in parameters of thyroid function (43). Additionally, it is important to consider that, besides Ni, people are simultaneously exposed to thousands of chemical substances (some still unexplored for their potential to endocrine disruption), which could also affect the level of thyroid hormones. The research study’s limitations include a small sample size with an inadequate distribution of healthy and unhealthy participants, a limited investigation of confounding factors within the population, and differences in the age of healthy and unhealthy participants that could potentially have influenced the obtained results. Amidst its shortcomings, the present study suggests the possibility of the dose-response relationship between Ni and changes in thyroid hormones/parameters of thyroid function, indicating that, especially in male population, even Ni doses higher than 0.397 and 1.36 µg/L might pose a 10% risk in the alterations of fT3 level and SPINA-GT, respectively.

These results highlight the possibility of applying the BMD approach in the examination of thyroid gland function. However, having in mind its limitations and lack of current knowledge considering the probable mechanism of Ni toxicity, especially regarding its endocrine effects, further investigation is needed. In light of this, our forthcoming animal research will explore whether low doses of toxic metals present in a mixture (extrapolated from human data) might have adverse effects in controlled circumstances.

## 5 Conclusion

In the current study, the existence of a dose-response relationship was established between Ni and every measured parameter of thyroid function in the investigated population from Belgrade, Serbia. However, most of the obtained evidence indicated possible connection between Ni exposure and higher risk of perturbation of thyroid function in male participants. Correlation was observed for Ni-SPINA-GT and Ni-fT4 pairs in men (negative correlation), while the narrowest BMD intervals were obtained also in men, for Ni - SPINA-GT (1.36-60.9 µg/L) and Ni - fT3 (0.397-66.8 µg/L) pair. Furthermore, the results of our study have indicated that even 83.25 and 78.68% of men in the investigated population were in 10% higher risk of Ni-induced fT3 and SPINA-GT alterations, respectively. In this study, the possible influence of Ni on the disorder of the secretory function of the thyroid gland was proven. Despite being challenged by several limitations which are inherent to research involving human subjects, the results of the present study are noteworthy enough to encourage further investigation of Ni-induced disturbances of thyroid function, especially in men, and highlight the value of utilizing the proposed BMD method in assessing human data.

## Data availability statement

The datasets for this study can be found in the Mendeley open data repository [https://data.mendeley.com/datasets/zbbgmvnz2r].

## Ethics statement

The studies involving human participants were reviewed and approved by o University Hospital “Bežanijska kosa” Medical Center’s Scientific and Ethical Committee (9740/3) o Clinical Center of Serbia’s Ethical Committee (526/9, 31/8, and 579/19) o Medical Faculty of the University of Belgrade (1322/XII-5) o University of Belgrade’s Ethical Commission for Biomedical Research (650/2 and 288/2). The patients/participants provided their written informed consent to participate in this study.

## Author contributions

DM and KB: Conceptualization, Formal analysis, Investigation, Writing - Original Draft. DJ: Formal analysis, Investigation, Data Curation. SR: Formal analysis, Data Analysis. MZ: Sample collection, Investigation. BA, DD-C and ZB: Methodology, Investigation, Formal analyses. AD: Conceptualization, Data Curation, Writing - Review and Editing, Supervision, Funding acquisition, Project administration. All authors contributed to the article and approved the submitted version.
